# Syncope With Sinus Arrest Following a Single Dose of a COVID-19 Vaccine

**DOI:** 10.7759/cureus.34309

**Published:** 2023-01-28

**Authors:** Mohamed Ghoweba, Muhie Dean Sabayon

**Affiliations:** 1 Internal Medicine, Texas A&M College of Medicine/CHRISTUS Good Shepherd Medical Center, Longview, USA; 2 Cardiac Electrophysiology, University of Texas Medical Branch, Galveston, USA

**Keywords:** cardiac electrophysiology, sars cov-2, covid-19, sinus arrest, pfizer-biontech covid-19 vaccine related adverse events, covid-19 vaccine related adverse events, vaccine induced bradycardia, syncope

## Abstract

Vaccines against the severe acute respiratory syndrome coronavirus 2(SARS-CoV-2) are of paramount importance in combating the current coronavirus disease 2019 (COVID-19) pandemic. Syncopal episodes following routine vaccinations are well-reported; however, only a few cases of syncope following SARS-CoV-2 vaccines exist in the literature. This is a case report of a 21-year-old female patient who developed recurrent syncopal attacks over three months that started one day after receiving the first dose of the Pfizer-BioNTech COVID-19 vaccine (Pfizer, New York City; BioNTech, Mainz, Germany). Holter monitoring during successive episodes showed progressive bradycardia followed by a prolonged sinus arrest. The patient eventually required pacemaker placement that resulted in the total resolution of her symptoms. Further studies are required to investigate a possible correlation and the mechanisms involved.

## Introduction

Vaccination against the severe acute respiratory syndrome coronavirus 2 (SARS-CoV-2) virus plays a pivotal role in the fight against the ongoing coronavirus disease 2019 (COVID-19) pandemic. While these vaccines have been proven to be predominantly safe and effective, adverse events have been reported with their use as with others [1]. Syncope is characterized by a transient loss of consciousness and postural tone secondary to a decrease in cerebral perfusion that is followed by spontaneous recovery. This is usually secondary to cardiac, orthostatic, neurocardiogenic, neurogenic, and cerebrovascular causes. Syncope-related falls can lead to serious sequelae including skull fractures, intracranial hemorrhages, and cerebral contusions [2]. Although vaccination-associated syncope has been well reported, few reports associated with the use of SARS-CoV-2 vaccines have been described. We report a case of recurrent syncopal episodes with a sinus arrest following the first dose of the Pfizer-BioNTech COVID-19 vaccine (Pfizer, New York City; BioNTech, Mainz, Germany).

## Case presentation

A 21-year-old Caucasian woman with a medical history of hypothyroidism secondary to Hashimoto’s thyroiditis, chronic migraine headaches without aura, and anxiety disorder presented to the outpatient electrophysiology clinic following three episodes of syncope within three months.

The patient endured her first episode one day following receiving her first dose of the Pfizer-BioNTech SARS-CoV-2 vaccine. She then had two other episodes that appeared to be cyclical in nature occurring every four weeks. The patient denied any heavy menstrual bleeding that could have explained her symptoms. She described having tunneled vision prior to losing consciousness; however, she denied any preceding palpitations, chest pain, dyspnea, diaphoresis, nausea, vomiting, or presyncope. She denied any preceding or coinciding headaches during her episodes. Upon regaining consciousness, she reported being fully awake and alert. Her episodes were witnessed with no reports of seizure-like activity involving tonic-clonic movements, tongue-biting, or urine or stool incontinence. She sustained head trauma during one of the episodes that required scalp sutures.

**Figure 1:**
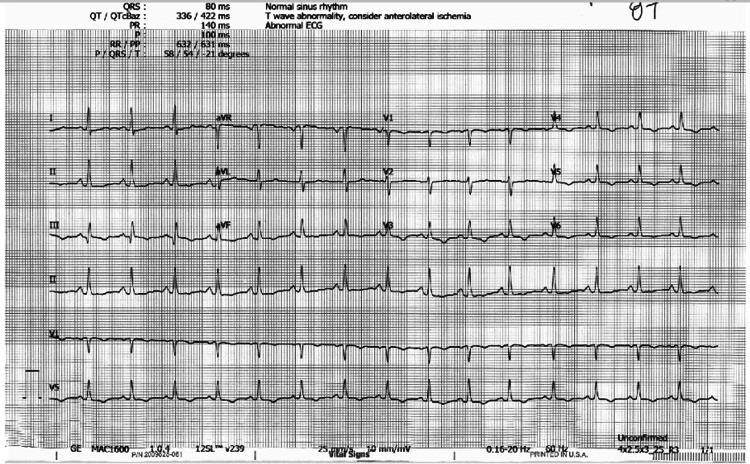
Baseline 12-lead electrocardiogram showing a normal sinus rhythm with T wave inversions on anterolateral leads. No other major abnormalities were noted.

The patient had a recent extensive workup that included basic metabolic, vitamin (vitamin B12), and thyroid (TSH, free T4) panels, which were all within the normal range. Upon presentation, the patient’s vital signs including orthostatic blood pressure, and physical exam were unremarkable. A 12-lead electrocardiogram showed a normal sinus rhythm without any evidence of manifest pre-excitation, epsilon waves, or any QT prolongation. T wave inversions were noted on anterolateral leads (Figure 1). The patient had been started on a transdermal hormonal patch for contraception four weeks prior to the first syncopal episode. She was advised to discontinue the patch due to an increased risk of thromboembolism given her history of migraines. Her syncopal attacks were deemed to be secondary to possible hormonal imbalances associated with her hormonal contraceptive. Nevertheless, she was placed on a two-week Holter monitor to rule out conduction abnormalities. An echocardiogram was ordered that revealed normal left ventricular function, normal wall motion, and no evidence of pericardial effusion.

**Figure 2:**
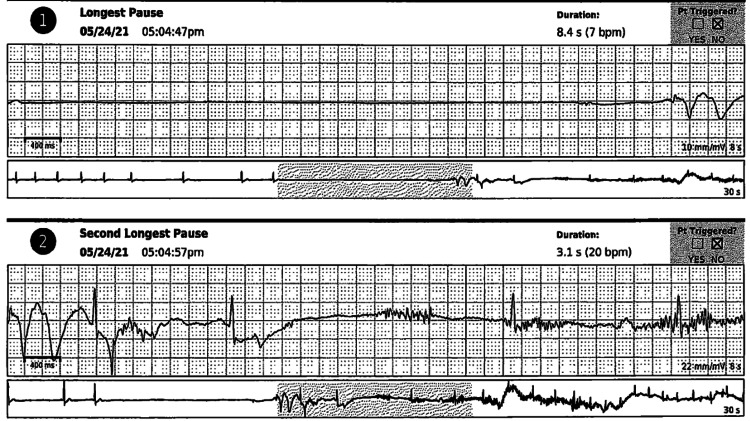
Holter monitor interrogation showing two prolonged pauses lasting 8.4 and 3.1 seconds

Five days later, the patient presented to the clinic following another episode of syncope one day prior. A 12-lead electrocardiogram revealed a normal sinus rhythm with no other abnormalities. Upon reviewing her monitor results, she was found to have been in a normal sinus rhythm that progressed to sinus bradycardia followed by a sinus arrest with no underlying escape for 8.4 seconds that correlated with her syncope. She then had a few beats of sinus bradycardia, followed by another 3.1-second pause of the same mechanism (Figure 2). Hence, a diagnosis of cardioinhibitory neurocardiogenic syncope was established. Treatment options were discussed with the patient and a decision was made to place a permanent dual-chamber pacemaker given her repeated syncopal attacks with a pause exceeding eight seconds and a related head injury. A pacemaker was placed and the patient remained syncope-free afterward.

Unfortunately, the patient presented to an outside facility a few days later with severe, sharp, left-sided chest pain that was positional in nature, worsening on lying down, and improving on leaning forward. No other associated symptoms were reported at that time. Pericarditis was suspected. To rule out other chest pathologies including pulmonary embolism, a chest computed tomography angiogram (CTA) was performed that revealed a right ventricular apical lead perforation. She was started on colchicine and ketorolac with a good response. The patient was discharged a day later on the same treatment course with an outpatient follow-up. She underwent a successful right ventricular lead extraction and re-insertion of a new lead four days later. The patient presented three months later with worsening exertional dyspnea that progressed to dyspnea at rest. She was saturating well on room air. D-dimer was elevated at 848 ng/mL. A CT scan following a pulmonary embolism protocol revealed a right pulmonary embolus (PE). Pericardial effusion was noted as well. The patient was admitted to the hospital. A workup was performed to determine an underlying etiology behind her pulmonary embolism including lupus anticoagulant profile, anti-neutrophilic cytoplasmic antibody, antinuclear antibody, phospholipid (cardiolipin) antibody IgG/IgA/IgM, C3 and C4 complement, and erythrocyte sedimentation rate, and was unremarkable. Hence, her PE was deemed secondary to her use of hormonal contraceptives that had been discontinued three months prior. C-reactive protein was slightly elevated at 1.41 mg/dL. The patient's pericardial effusion was completely drained yielding a bloody pericardial fluid. A pericardial drain was left in place. Pericardial fluid analysis showed reactive mesothelial cells, pigmented macrophages, and chronic inflammation. Given the minimal output via the drain, it was eventually removed three days into hospitalization. No anti-inflammatory medications were deemed necessary at that time. The patient was started on anticoagulation with apixaban 5 mg twice daily for her PE upon discharge. A follow-up chest CTA three months following the detection of her PE showed total resolution of the right pulmonary embolus with no evidence of pericardial effusion. Apixaban was therefore discontinued after completing a three-month course.

Upon follow-up over the next two months, the patient did not report further syncopal attacks and had no complaints. A repeat transthoracic echocardiogram (TTE) did not reveal any pericardial effusions with normal left ventricular function.

## Discussion

Differential diagnoses for this patient encompassed different causes of syncope including orthostatic hypotension, and of neurogenic, cardiac, and neurocardiogenic etiology. Orthostatic hypotension was ruled out given the patient's normal orthostatic blood pressure. The patient did not exhibit any neurological symptoms preceding or coinciding with her syncopal attacks with no reported headache, blurred vision, numbness, tingling, weakness, slurred speech, tonic-clonic contractions, tongue-biting, or loss of control over stool or urine. Cardiac causes of syncope mainly include inherited channelopathies such as Brugada syndrome, paroxysmal atrioventricular blocks, and hypertrophic obstructive cardiomyopathy. However, the lack of characteristic features of channelopathies on electrocardiogram, and the absence of evidence of structural heart disease on echocardiography excluded cardiac causes. Hence, given the patient's syncopal features, a diagnosis of cardioinhibitory reflex syncope was made.

SARS-CoV-2 vaccines are of paramount importance in combating the current COVID-19 pandemic. Despite their proven efficacy and safety, various adverse events have been associated with the use of these vaccines [1]. Syncopal episodes following routine vaccinations, including the human papilloma virus (HPV), quadrivalent meningococcal conjugate vaccine (MCV4), tetanus toxoid (TT), and reduced diphtheria toxoid, acellular pertussis (Tdap) vaccines, are well documented [3-7]. However, only a few cases of COVID-19-vaccine-associated syncope exist in the literature [8-11]. Important to note is that a higher incidence of reported vaccine-associated syncope was found in younger female populations under the age of 20. The majority of episodes occurred between 5 and 15 minutes following administration [5,6].

Neurocardiogenic syncope, also known as vasovagal syncope (VVS), is caused by an abnormal autonomic response to various stimuli including hypovolemia, dehydration, pain, and emotion, among others. It involves the activation of cardiac C fibers with a resulting change in the heart rate or vascular tone; however, the exact mechanism is yet to be elucidated. Two types of VVS exist: a cardioinhibitory response associated with bradycardia as in this case, and a vasodepressor response associated with hypotension [12]. The pathophysiological mechanism underlying vaccine-associated syncope remains ambiguous, although it has been mostly attributed to a vasovagal reaction. Fear and anxiety have been postulated as triggers and are of possible significance with COVID-19 vaccinations, given the novel nature of these vaccines [10,12]. There have been reports of delayed cardioinhibitory responses following the SARS-CoV-2 vaccines [8]. QT prolongation related to COVID-19-vaccine-associated myocarditis has been implicated as well; however, myocarditis has been excluded in this case [10]. To the best of our knowledge, the recurrent nature of this patient’s syncope as well as the associated prolonged pauses requiring pacemaker placement have not been reported in the literature before.

It is important to note that the patient’s history of anxiety might have been a confounding factor. Other confounding factors including heavy menstrual bleeding leading to orthostatic hypotension were not evident in this case. The case sheds the light on recurrent neurocardiogenic syncope as a possible adverse event following the novel mRNA Pfizer-BioNTech COVID-19 vaccine.

## Conclusions

Syncope should be considered as a possible side effect of the novel COVID-19 vaccinations. Although multiple mechanisms have been postulated, the exact etiology remains to be clear. Episodes can be recurrent and involve prolonged pauses that warrant pacemaker placement to prevent serious sequelae. Further studies are warranted to investigate a possible correlation and the mechanisms involved.
